# Induction of Suicidal Erythrocyte Death by Cantharidin

**DOI:** 10.3390/toxins7082822

**Published:** 2015-07-28

**Authors:** Kousi Alzoubi, Jasmin Egler, Marilena Briglia, Antonella Fazio, Caterina Faggio, Florian Lang

**Affiliations:** 1 Department of Physiology, University of Tuebingen, Gmelinstr. 5, Tuebingen 72076, Germany; E-Mails: kossai.z@gmail.com (K.A.); jasmin.egler@t-online.de (J.E.); 2 Department of Biological and Environmental Sciences, University of Messina, Viale Ferdinando Stagno d'Alcontres 31, S. Agata-Messina 98166, Italy; E-Mails: marilenabriglia@icloud.com (M.B.); Antonellafazio89@gmail.com (A.F.); cfaggio@unime.it (C.F.)

**Keywords:** phosphatidylserine, calcium, cell volume, staurosporine, kinase, eryptosis

## Abstract

The natural phosphoprotein phosphatase inhibitor cantharidin, primarily used for topical treatment of warts, has later been shown to trigger tumor cell apoptosis and is thus considered for the treatment of malignancy. Similar to apoptosis of tumor cells, erythrocytes may undergo eryptosis, a suicidal cell death characterized by cell shrinkage and translocation of cell membrane phosphatidylserine to the erythrocyte surface. Signaling of eryptosis includes increase of cytosolic Ca^2+^-activity ([Ca^2+^]_i_), ceramide, oxidative stress and dysregulation of several kinases. Phosphatidylserine abundance at the erythrocyte surface was quantified utilizing annexin-V-binding, cell volume from forward scatter, [Ca^2+^]_i_ from Fluo3-fluorescence, ceramide from antibody binding, and reactive oxidant species (ROS) from 2′,7′-dichlorodihydrofluorescein diacetate (DCFDA) fluorescence. A 48 h treatment of human erythrocytes with cantharidin significantly increased the percentage of annexin-V-binding cells (≥10 μg/mL), significantly decreased forward scatter (≥25 μg/mL), significantly increased [Ca^2+^]_i_ (≥25 μg/mL), but did not significantly modify ceramide abundance or ROS. The up-regulation of annexin-V-binding following cantharidin treatment was not significantly blunted by removal of extracellular Ca^2+^ but was abolished by kinase inhibitor staurosporine (1 μM) and slightly decreased by p38 inhibitor skepinone (2 μM). Exposure of erythrocytes to cantharidin triggers suicidal erythrocyte death with erythrocyte shrinkage and erythrocyte membrane scrambling, an effect sensitive to kinase inhibitors staurosporine and skepinone.

## 1. Introduction

Cantharidin, a traditional Chinese natural product, has been successfully used for the treatment of warts, molluscum contagiosum, and callus removal [1]. Cantharidin and its demethylated analogue norcantharidin have more recently been shown to be effective against malignancy [2,3,4]. The anticancer effects are in part attributed to their inhibitory effect on phosphoprotein phosphatases [4,5] and stimulation of tumor cell apoptosis [6,7,8,9]. Further mechanisms invoked in (nor) cantheridine induced apoptosis include mitochondrial dysregulation [10,11], cytosolic cytochrome c release [3], activation of caspase-9 [3,12], induction of oxidative stress [10,13] and activation of the transcription factor p53 with subsequent triggering of p53 dependent gene expression [13,14].

Erythrocytes lack mitochondria and nuclei, but are nevertheless able to enter suicidal cell death or eryptosis, which is characterized by cell shrinkage [15] and translocation of phosphatidylserine to the outer surface of the erythrocyte cell membrane [16]. Signaling involved in the stimulation of eryptosis includes increased cytosolic Ca^2+^ activity ([Ca^2+^]_i_), ceramide [17], oxidative stress [16], caspase activation [16,18,19], activation of casein kinase 1α, Janus-activated kinase JAK3, protein kinase C, p38 kinase, and PAK2 kinase [16] or inhibition of AMP activated kinase AMPK, cGMP-dependent protein kinase, and sorafenib and sunitinib sensitive kinases [16]. Eryptosis is triggered by a wide variety of xenobiotics [16,20,21,22,23,24,25,26,27,28,29,30,31,32,33,34,35,36,37,38,39,40,41,42].

The present study explored whether cantharidin stimulates eryptosis. To this end, erythrocytes from healthy volunteers were exposed to cantharidin, phosphatidylserine abundance at the erythrocyte surface determined using annexin-V-binding and cell volume estimated from forward scatter in flow cytometry. Moreover, [Ca^2+^]_i_ was estimated utilizing Fluo3-fluorescence, ceramide abundance utilizing specific antibodies, and abundance of reactive oxidant species utilizing 2′,7′-dichlorodihydrofluorescein diacetate (DCFDA) fluorescence. The involvement of phosphorylation was tested utilizing kinase inhibitors staurosporine and skepinone.

## 2. Results and Discussion

The present study explored whether cantharidin is capable to trigger eryptosis, the suicidal death of erythrocytes, *i.e.*, of cells lacking mitochondria and nuclei, organelles considered to play a major role in the triggering of apoptosis. Hallmarks of eryptosis are phosphatidylserine translocation to the cell surface and cell shrinkage.

**Figure 1:**
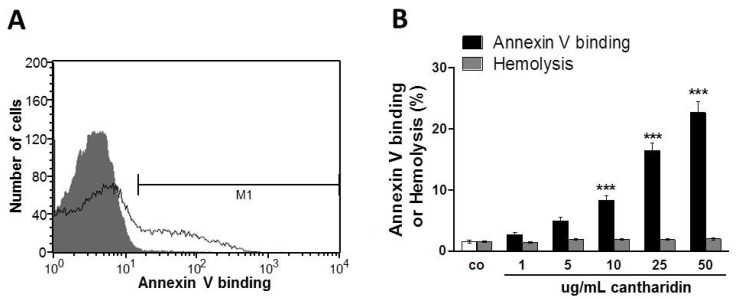
Effect of cantharidin on phosphatidylserine exposure. (**A**) Original histogram of annexin-V-binding of erythrocytes following exposure for 48 h to Ringer solution without (grey area) and with (black line) presence of 50 μg/mL cantharidin. M1 indicates the annexin-V-fluorescence defining the percentage of annexin-V-binding erythrocytes. (**B**) Arithmetic means ± SEM of erythrocyte annexin-V-binding (n = 12) following incubation for 48 h to Ringer solution without (white bar) or with (black bars) presence of cantharidin (1–50 μg/mL). For comparison, the effect of cantharidin on hemolysis is shown (grey bars). *** (*p* < 0.001) indicates significant difference from the absence of cantharidin (ANOVA).

Phosphatidylserine at the cell surface was detected utilizing binding of FITC-labeled annexin-V to phosphatidylserine. The abundance of FITC-labeled annexin-V was determined by flow cytometry. As illustrated in Figure 1, a 48 h exposure to cantharidin enhanced the percentage of annexin-V-binding erythrocytes, an effect reaching statistical significance at 10 μg/mL cantharidin concentration. As shown in Figure 1, cantharidin did not modify hemolysis.

**Figure 2:**
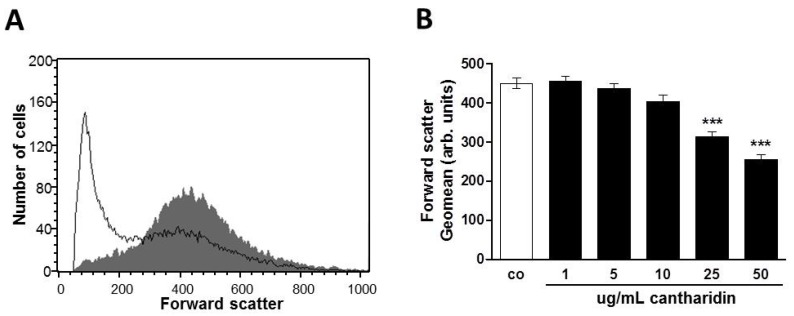
Effect of cantharidin on erythrocyte forward scatter: (**A**) Original histogram of forward scatter of erythrocytes following exposure for 48 h to Ringer solution without (grey area) and with (black line) presence of 50 μg/mL cantharidin. (**B**) Arithmetic means ± SEM (n = 12) of the geometric mean erythrocyte forward scatter (FSC) following incubation for 48 h to Ringer solution without (white bar) or with (black bars) cantharidin (1–50 μg/mL). *** (*p <* 0.001) indicate significant difference from the absence of cantharidin (ANOVA).

Forward scatter was determined in flow cytometry as a measure of erythrocyte cell volume. As shown in Figure 2, a 48 h cantharidin treatment was followed by a decrease of erythrocyte forward scatter, an effect reaching statistical significance at 25 μg/mL cantharidin concentration.

**Figure 3:**
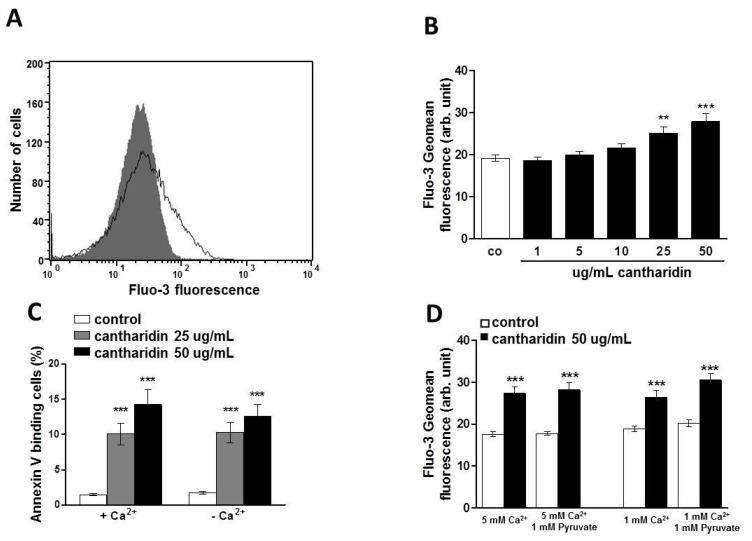
Effect of cantharidin on erythrocyte Ca^2+^ activity and Ca^2+^ sensitivity of cantharidin-induced phosphatidylserine exposure: (**A**) Original histogram of Fluo3 fluorescence in erythrocytes following exposure for 48 h to Ringer solution without (grey area) and with (black line) presence of cantharidin (50 μg/mL). (**B**) Arithmetic means ± SEM (n = 12) of the Fluo3 fluorescence (arbitrary units) in erythrocytes exposed for 48 h to Ringer solution without (white bar) or with (black bars) cantharidin (1–50 μg/mL). (**C**) Arithmetic means ± SEM (n = 20) of annexin-V-binding of erythrocytes after a 48 h treatment with Ringer solution without (white bars) or with 25 μg/mL (grey bars) or 50 μg/mL (black bars) cantharidin in the presence (left bars, +Ca^2+^) and absence (right bars, −Ca^2+^) of Ca^2+^. ** (*p <* 0.01) *** (*p <* 0.001) indicate significant difference from the absence of cantharidin (ANOVA). (**D**) Arithmetic means ± SEM (n = 9) of the Fluo3 fluorescence (arbitrary units) in erythrocytes exposed for 48 h to Ringer solution without (white bar) or with (black bars) cantharidin (50 μg/mL) and stained with Fluo3 AM in Ringer solution with (left bars) 5 mM CaCl_2_ ± 1 mM sodium pyruvate, or with (right bars) 1 mM CaCl_2_ ± 1 mM sodium pyruvate. *** (*p <* 0.001) indicate significant difference from the absence of cantharidin (ANOVA).

Both phospholipid scrambling of the erythrocyte membrane and cell shrinkage could be triggered by activation of Ca^2+^ permeable cation channels with subsequent Ca^2+^ entry. Fluo3 fluorescence was thus employed to test whether cantharidin influences cytosolic Ca^2+^ activity ([Ca^2+^]_i_). As illustrated in Figure 3A,B, a 48 h exposure to cantharidin increased the Fluo3 fluorescence, an effect requiring 25 μg/mL cantharidin concentration for statistical significance. To test the effect of calcium concentration in the staining solution while loading with Fluo3 and to test the potential toxic effects from released formaldehyde as a byproduct of esterification [43,44], we treated erythrocytes for 48 h with Ringer solution without or with cantharidin (50 μg/mL) and then stained for 30 min with Fluo3 AM in Ringer solution containing 1 or 5 mM CaCl_2_ in the presence and absence of 1 mM sodium pyruvate.

As illustrated in Figure 3D, the stimulatory effect of cantharidin on Fluo3 staining, in the presence of 1 or 5 mM CaCl_2_, was similar in the presence or absence of pyruvate. A further series of experiments explored whether cantharidin-induced translocation of phosphatidylserine to the cell surface required entry of extracellular Ca^2+^. To this end, erythrocytes were incubated for 48 h in the absence or presence of 25 or 50 μg/mL cantharidin, both in the presence or nominal absence of extracellular Ca^2+^. As illustrated in Figure 3C, removal of extracellular Ca^2+^ did not significantly blunt the effect of cantharidin on annexin-V-binding. Instead, cantharidin significantly increased the percentage of annexin-V-binding erythrocytes to similarly high levels in the absence and in the presence of extracellular Ca^2+^. Thus, triggering of eryptosis did not require entry of extracellular Ca^2+^.

Eryptosis could be stimulated independently from increased [Ca^2+^]_i_ by ceramide. Thus, specific antibodies were utilized to quantify ceramide abundance at the erythrocyte surface. As a result, the ceramide abundance was similar following a 48 h incubation in the absence of cantharidin (11.3 ± 1.3 a.u., n = 9), presence of 10 μg/mL cantharidin (11.1 ± 1.4 a.u., n = 9) and presence of 50 μg/mL cantharidin (11.2 ± 1.5 a.u., *n* = 9). Thus, cantharidin did not enhance ceramide abundance.

**Figure 4:**
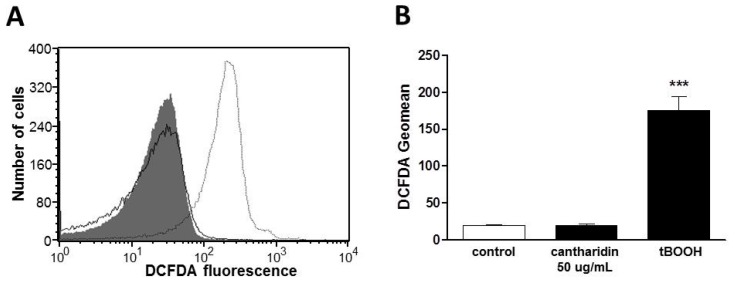
Effect of cantharidin on reactive oxygen species: (**A**) Original histogram of 2′,7′-dichlorodihydrofluorescein diacetate (DCFDA) fluorescence in erythrocytes following exposure for 48 h to Ringer solution without (grey shadow) or with (black line) the presence of 50 μg/mL cantharidin or, for comparison, following a one hour exposure to 100 μM tert-butyl-hydroperoxide (tBOOH, light grey line). (**B**) Arithmetic means ± SEM (n = 4) of the erythrocyte DCFDA fluorescence following incubation for 48 h to Ringer solution without (control, white bar) or with presence of 50 μg/mL cantharidin (black bar) or for one hour with presence of 100 μM tBOOH (black bar). *** (*p <* 0.001) indicates significant difference from the absence of cantharidin and tBOOH (ANOVA).

Additional experiments explored whether cantharidin influences the formation of reactive oxygen species (ROS). To this end, ROS was quantified utilizing 2′,7′-dichlorodihydrofluorescein diacetate (DCFDA). As shown in Figure 4, a 48 h exposure to cantharidin (50 μg/mL) did not modify DCFDA fluorescence. As a positive control, a 48 h exposure to tert-butyl-hydroperoxide (100 μM, tBOOH) was followed by a strong and highly significant increase of DCFDA fluorescence (Figure 4).

**Figure 5:**
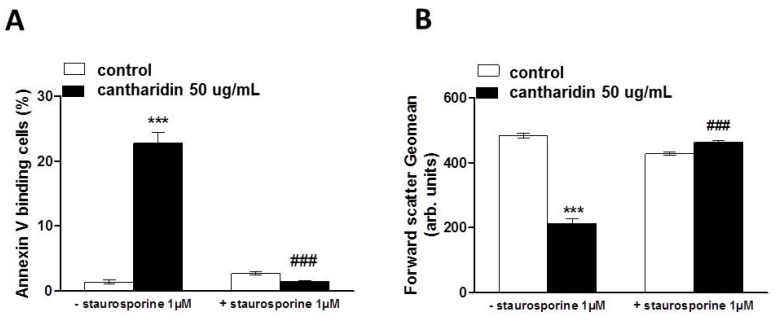
Effect of cantharidin on phosphatidylserine exposure and forward scatter in the absence and presence of staurosporine: (**A**) Arithmetic means ± SEM (n = 12) of annexin-V-binding of erythrocytes after a 48 h treatment with Ringer solution without (white bars) or with (black bars) 50 μg/mL cantharidin in the absence (left bars, −Staurosporine) and presence (right bars, +Staurosporine) of kinase inhibitor staurosporine (1 μM). (**B**) Arithmetic means ± SEM (n = 8) of forward scatter of erythrocytes after a 48 h treatment with Ringer solution without (white bars) or with (black bars) 50 μg/mL cantharidin in the absence (left bars, −Staurosporine) and presence (right bars, +Staurosporine) of kinase inhibitor staurosporine (1 μM). *** (*p <* 0.001) indicates significant difference from the absence of cantharidin, **###** (*p <* 0.001) indicates significant difference from the respective value in the absence of staurosporine.

In order to test whether the effect of cantharidin was related to protein phosphorylation, the effect of cantharidin on translocation of phosphatidylserine to the cell surface was determined in the absence and presence of protein kinase inhibitor staurosporine (1 μM). Erythrocytes were incubated for 48 h in the absence or presence of 50 μg/mL cantharidin, both in the absence or presence of staurosporine (1 μM). Addition of staurosporine fully abrogated the effect of cantharidin on annexin-V-binding (Figure 5A) and abrogated the effect of cantharidin on forward scatter (Figure 5B).

**Figure 6:**
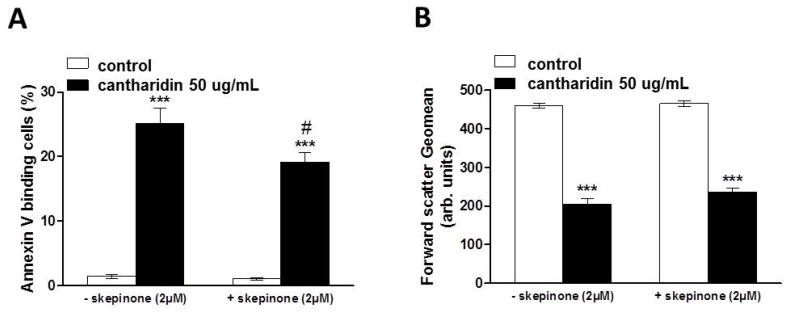
Effect of cantharidin on phosphatidylserine exposure and forward scatter in the absence and presence of skepinone: (**A**) Arithmetic means ± SEM (n = 12) of annexin-V-binding of erythrocytes after a 48 h treatment with Ringer solution without (white bars) or with (black bars) 50 μg/mL cantharidin in the absence (left bars, −skepinone) and presence (right bars, +skepinone) of p38 kinase inhibitor skepinone (2 μM). (**B**) Arithmetic means ± SEM (n = 12) of forward scatter of erythrocytes after a 48 h treatment with Ringer solution without (white bars) or with (black bars) 50 μg/mL cantharidin in the absence (left bars, −skepinone) and presence (right bars, +skepinone) of kinase inhibitor skepinone (2 μM). *** (*p <* 0.001) indicates significant difference from the absence of cantharidin, **#** (*p <* 0.05) indicates significant difference from the respective value in the absence of skepinone.

Additional experiments explored whether cantharidin effect was influenced by the protein kinase inhibitor skepinone. Erythrocytes were incubated for 48 h in the absence or presence of 50 μg/mL cantharidin, both in the absence or presence of skepinone (2 μM). Addition of skepinone slightly inhibited the effect of cantharidin on annexin-V-binding, but did not inhibit the effect of cantharidin on forward scatter (Figure 6).

The present observations reveal a novel effect of cantharidin, *i.e.*, the triggering of erythrocyte shrinkage and erythrocyte cell membrane scrambling with phosphatidylserine translocation from the cell interior to the erythrocyte surface. Cell membrane scrambling and cell shrinkage are the hallmarks of eryptosis, the suicidal erythrocyte death. In contrast to its effect on eryptosis, cantharidin did not induce appreciable hemolysis. The cantharidin concentration required for stimulation of erythrocyte cell membrane scrambling (10 μg/mL) was in the range of cantharidin concentrations required to elicit tumor cell apoptosis (IC50 ≈ 20 μM ≈ 4 μg/mL) [7] but higher than those reported in rats (~0.1 μg/mL) [45] and dogs (~0.15 μg/mL) [46]. At higher concentrations, cantharidin increases cytosolic Ca^2+^ activity ([Ca^2+^]_i_), which may contribute to the triggering of cell shrinkage by activation of Ca^2+^ sensitive K^+^ channels, K^+^ exit, cell membrane hyperpolarization, Cl^−^ exit and thus cellular loss of KCl with osmotically obliged water [15].

Removal of extracellular Ca^2+^ did, however, not appreciably blunt the stimulation of annexin-V-binding following cantharidin treatment. Even in the absence of extracellular Ca^2+^ cantharidin significantly enhanced the phosphatidylserine abundance at the cell surface. Thus, the effect of cantharidin on phosphatidylserine translocation did not require Ca^2+^ entry. The observation that cantharidin-induced eryptosis occurred even in the absence of extracellular Ca^2+^ cannot be taken as evidence that cantharidin was without effect on Ca^2+^ entry. As a matter of fact, cantharidin did increase [Ca^2+^]_i_, an effect presumably due to stimulation of Ca^2+^ entry. Moreover, the increase of [Ca^2+^]_i_ following cantharidin treatment could have contributed to stimulation of eryptosis. However, cantharidin treatment was able to trigger eryptosis even in the absence of Ca^2+^ entry, an observation pointing to the operation of a Ca^2+^ insensitive mechanism. Such a mechanism could have been ceramide. However, the cantharidin-induced eryptosis does apparently not involve ceramide formation or translocation.

Cantharidin further did not trigger oxidative stress, a known stimulator of eryptosis [16]. In nucleated cells, cantharidin does induce oxidative stress [10,13], an effect presumably requiring mitochondria [10,11] and thus lacking in erythrocytes.

Instead, the effect of cantharidin on both, cell membrane scrambling and cell volume was apparently related to its inhibitory effect on phosphoprotein phosphatases [4,5]. The effect of cantharidin on both, cell membrane scrambling and cell volume, was completely abrogated by the kinase inhibitor staurosporine. It is tempting to speculate that cantharidin prevents dephosphorylation of target proteins, which are phosphorylated by staurosporine sensitive kinases. Regulators of erythrocyte cell volume include KCl symport on the one hand and Na^+^,K^+^,2Cl^−^ cotransport on the other, carriers under the control of several kinases [47,48,49]. Thus, deranged activity of those kinases or carriers could contribute to cell volume loss. Cell shrinkage and loss of cellular K^+^ in turn fosters cell membrane scrambling [15]. The sensitivity of cantharidin induced eryptosis to staurosporine and skepinone indeed suggests a role of kinases in the triggering of eryptosis. Staurosporine completely abrogated the effect of cantharidin on both, phosphatidylserine translocation and cell volume, whereas skepinone only slightly blunted the effect of cantharidin on phosphatidylserine translocation and did not appreciably modify the cantharidin-induced cell shrinkage. Apparently, p38 kinase contributes to but does not account for cantharidine-induced eryptosis. Clearly, additional experimental effort is required to dissect the specific kinase(s) and phosphatase(s) involved in the cantharidin sensitive regulation of eryptosis.

The stimulation of eryptosis by cantharidin may add to the toxicity of the substance. Phosphatidylserine exposing erythrocytes are engulfed by macrophages and thus rapidly cleared from circulating blood and stimulation of eryptosis may thus lead to anemia [16]. Moreover, erythrocytes exposing phosphatidylserine at their surface may adhere to endothelial cells of the vascular wall [50], stimulate blood clotting and induce thrombosis [51,52,53]. Accordingly, stimulating phosphatidylserine exposure of erythrocytes may impede microcirculation [17,51,54,55,56,57]. The toxic effect may be augmented in clinical conditions associated with enhanced eryptosis, such as malignancy, hepatic failure, diabetes, uremia, hemolytic uremic syndrome, sepsis, fever, dehydration, mycoplasma infection, malaria, iron deficiency, sickle cell anemia, thalassemia, glucose-6-phosphate dehydrogenase deficiency, and Wilson’s disease [16].

## 3. Experimental Section

### 3.1. Erythrocytes, Solutions and Chemicals

Fresh Lithium-Heparin-anticoagulated blood samples were kindly provided by the blood bank of the University of Tübingen. The study was approved by the ethics committee of the University of Tübingen (184/2003 V). The blood was centrifuged at 120 g for 20 min at 23 °C and the platelets and leukocytes-containing supernatant was disposed. Erythrocytes were incubated *in vitro* for 48 h at a hematocrit of 0.4% in Ringer solution containing (in mM) 125 NaCl, 5 KCl, 1 MgSO_4_, 32 *N*-2-hydroxyethylpiperazine-*N*-2-ethanesulfonic acid (HEPES), 5 glucose, and 1 CaCl_2_; the pH was adjusted to 7.4 and the temperature kept at 37 °C. Where indicated, erythrocytes were exposed to cantharidin (Sigma Aldrich, Hamburg, Germany).

### 3.2. Annexin-V-Binding and Forward Scatter

After incubation under the respective experimental condition, a 150 μL cell suspension was washed in Ringer solution containing 5 mM CaCl_2_ and then stained with Annexin-V-FITC (1:200 dilution; ImmunoTools, Friesoythe, Germany) in this solution at 37 °C for 20 min under protection from light. In the following, the geometric mean of the forward scatter (FSC) was determined, and annexin-V fluorescence intensity was measured with an excitation wavelength of 488 nm and an emission wavelength of 530 nm on a FACS Calibur (BD, Heidelberg, Germany). In some experiments erythrocytes were preincubated in Ca^2+^ free solution. For determination of annexin-V-binding, addition of Ca^2+^ was required during the 15 min incubation with FITC-annexin-V. Immediately thereafter measurements were done so that the exposure to Ca^2+^ was too short to trigger significant phosphatidylserine translocation.

### 3.3. Hemolysis

For the determination of hemolysis, the samples were centrifuged (3 min at 1600 rpm, room temperature) after incubation under the respective experimental conditions and the supernatants were harvested. As a measure of hemolysis, the hemoglobin (Hb) concentration of the supernatant was determined photometrically at 405 nm. The absorption of the supernatant of erythrocytes lysed in distilled water was defined as 100% hemolysis.

### 3.4. Intracellular Ca^2+^

After incubation, a 150 μL cell suspension was washed in Ringer solution and then loaded with Fluo-3/AM (Biotium, Hayward, CA, USA) in Ringer solution containing 5 mM CaCl_2_ and 5 μM Fluo-3/AM. The cells were incubated at 37 °C for 30 min and washed twice in Ringer solution containing 5 mM CaCl_2_. The Fluo-3/AM-loaded erythrocytes were resuspended in 200 μL Ringer. Then, Ca^2+^-dependent fluorescence intensity was measured with an excitation wavelength of 488 nm and an emission wavelength of 530 nm on a FACS Calibur.

### 3.5. Reactive Oxidant Species (ROS)

Oxidative stress was determined utilizing 2′,7′-dichlorodihydrofluorescein diacetate (DCFDA). After incubation, a 150 μL suspension of erythrocytes was washed in Ringer solution and then stained with DCFDA (Sigma, Schnelldorf, Germany) in Ringer solution containing DCFDA at a final concentration of 10 μM. Erythrocytes were incubated at 37 °C for 30 min in the dark and then washed three times in Ringer solution. The DCFDA-loaded erythrocytes were resuspended in 200 μL Ringer solution, and ROS-dependent fluorescence intensity was measured at an excitation wavelength of 488 nm and an emission wavelength of 530 nm on a FACS Calibur (BD).

### 3.6. Ceramide Abundance

For the determination of ceramide, a monoclonal antibody-based assay was used. After incubation, cells were stained for 1 h at 37 °C with 1 μg/mL anti ceramide antibody (clone MID 15B4, Alexis, Grünberg, Germany) in PBS containing 0.1% bovine serum albumin (BSA) at a dilution of 1:10. The samples were washed twice with PBS-BSA. Subsequently, the cells were stained for 30 min with polyclonal fluorescein isothiocyanate (FITC) conjugated goat anti-mouse IgG and IgM specific antibody (Pharmingen, Hamburg, Germany) diluted 1:50 in PBS-BSA. Unbound secondary antibody was removed by repeated washing with PBS-BSA. The samples were then analyzed by flow cytometric analysis with an excitation wavelength of 488 nm and an emission wavelength of 530 nm.

### 3.7. Statistics

Data are expressed as arithmetic means ± SEM. As indicated in the figure legends, statistical analysis was made using ANOVA with Tukey’s test as post-test and *t* test as appropriate. n denotes the number of different erythrocyte specimens studied. Since different erythrocyte specimens used in distinct experiments are differently susceptible to triggers of eryptosis, the same erythrocyte specimens have been used for control and experimental conditions.

## 4. Conclusions

Cantharidin stimulates erythrocyte cell membrane scrambling and cell shrinkage, both hallmarks of eryptosis, the suicidal erythrocyte death. The effect of cantharidin on cell membrane scrambling and cell shrinkage is abrogated by kinase inhibitor staurosporine and may thus be due to the known inhibitory effect of cantharidin on protein phosphatases.
